# (*E*)-2-{[(2-Amino­phen­yl)imino]­meth­yl}-5-(benz­yl­oxy)phenol and (*Z*)-3-benz­yl­oxy-6-{[(5-chloro-2-hy­droxy­phen­yl)amino]­methyl­idene}cyclo­hexa-2,4-dien-1-one

**DOI:** 10.1107/S2056989018005662

**Published:** 2018-04-27

**Authors:** Nadir Ghichi, Ali Benboudiaf, Chawki Bensouici, Yacine DJebli, Hocine Merazig

**Affiliations:** a Unit of Research CHEMS, University of Constantine 1, Algeria; b Biotechnology Research Center, Constantine, Algeria; c Laboratory of Materials Chemistry,University of Constantine 1, Algeria

**Keywords:** crystal structure, Schiff base, CUPRAC, anti­oxidant capacity, DFT calculations

## Abstract

The title Schiff base compounds, C_20_H_18_N_2_O_2_ (I) and C_20_H_16_ClNO_3_ (II), were synthesized from 4-benz­yloxy-2-hy­droxy­benzaldehyde by reaction with 1,2-di­amino­benzene for (I), and condensation with 2-amino-4-chloro­phenol for (II). Compound (I) adopts the enol–imine tautomeric form with an *E* configuration about the C=N imine bond. In contrast, the *o*-hy­droxy Schiff base (II), is in the keto–imine tautomeric form with a *Z* configuration about the CH—NH bond. Neither mol­ecule is planar. In (I), the central benzene ring makes dihedral angles of 46.80 (10) and 78.19 (10)° with the outer phenyl­amine and phenyl rings, respectively, while for (II), the corresponding angles are 5.11 (9) and 58.42 (11)°, respectively. The mol­ecular structures of both compounds are affected by the formation of intra­molecular contacts, an O—H⋯N hydrogen bond for (I) and an N—H⋯O hydrogen bond for (II); each contact generates an *S*(6) ring motif. In the crystal of (I), strong N—H⋯O hydrogen bonds form zigzag chains of mol­ecules along the *b-*axis direction. Mol­ecules are further linked by C—H⋯π inter­actions and offset π–π contacts and these combine to form a three-dimensional network. The density functional theory (DFT) optimized structure of compound (II), at the B3LYP/6–311+G(*d*) level, confirmed that the keto tautomeric form of the compound, as found in the structure determination, is the lowest energy form. The anti­oxidant capacities of both compounds were determined by the cupric reducing anti­oxidant capacity (CUPRAC) process.

## Abstract 2

The synthesis and structures of (*E*)-2-{[(2-amino­phen­yl)imino]­meth­yl}-5-(benz­yloxy)phenol (I) and (*Z*)-3-benz­yloxy-6-{[(5-chloro-2-hy­droxy­phen­yl)amino]­methyl­idene}cyclo­hexa-2,4-dien-1-one (II) are reported. The crystal structures of the mol­ecules are stabilized by N—H⋯O, O—H⋯O and C—H⋯π contacts. DFT calculations on the structure of (II) support the Keto–imine tautomeric form found in the solid state structure. The anti­oxident properties of both mol­ecules are investigated.

## Chemical context   

Schiff base compounds have been used as fine chemicals and medicinal substrates (Fun *et al.*, 2011 ▸). Studies of the tautom­erism of Schiff bases (Alpaslan *et al.*, 2011 ▸; Blagus *et al.*, 2010 ▸; Ünver *et al.*, 2002 ▸) have demonstrated that the stabilization of the keto–amino tautomer in the crystal depends mostly on the parent *o*-hydroxyl aldehyde, the type of the N-substituent, the electron withdrawing or donating of the N-substituent, its position and stereochemistry (Blagus *et al.*, 2010 ▸). Schiff base compounds exhibit a broad range of biological activities, including anti­fungal and anti­bacterial (da Silva *et al.*, 2011 ▸). They are used as anion sensors (Dalapati *et al.*, 2011 ▸; Khalil *et al.*, 2009 ▸), non-linear optical compounds (Sun *et al.*, 2012 ▸), and as versatile ligands in coordination chemistry (Khanmohammadi *et al.*, 2009 ▸; Keypour *et al.*, 2010 ▸). In view of the inter­est in such materials we have synthesized the title compounds, (I) and (II), and report their crystal structures here. The common structural feature of these compounds is the presence of a benz­yloxy substituent on the central ring, although each mol­ecule adopts a different tautomeric form. Density functional theory (DFT) calculations on (II), carried out at the B3LYP/6-311+G(d) level, are compared with the experimentally determined mol­ecular structure and confirm that the keto tautomeric form of this compound, similar to that found in the structure determination, is the lowest energy form. The anti­oxidant capacity of both compounds was determined by the cupric reducing anti­oxidant capacity (CUPRAC) process.
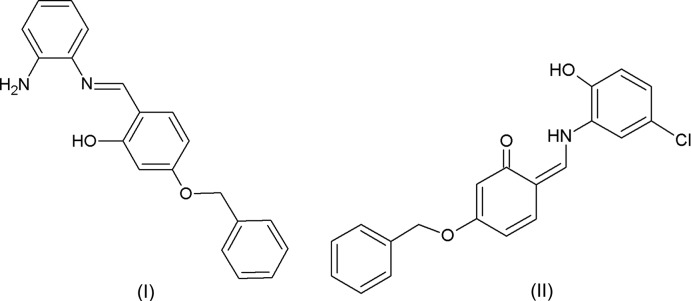


## Structural commentary   

**Figure 1:**
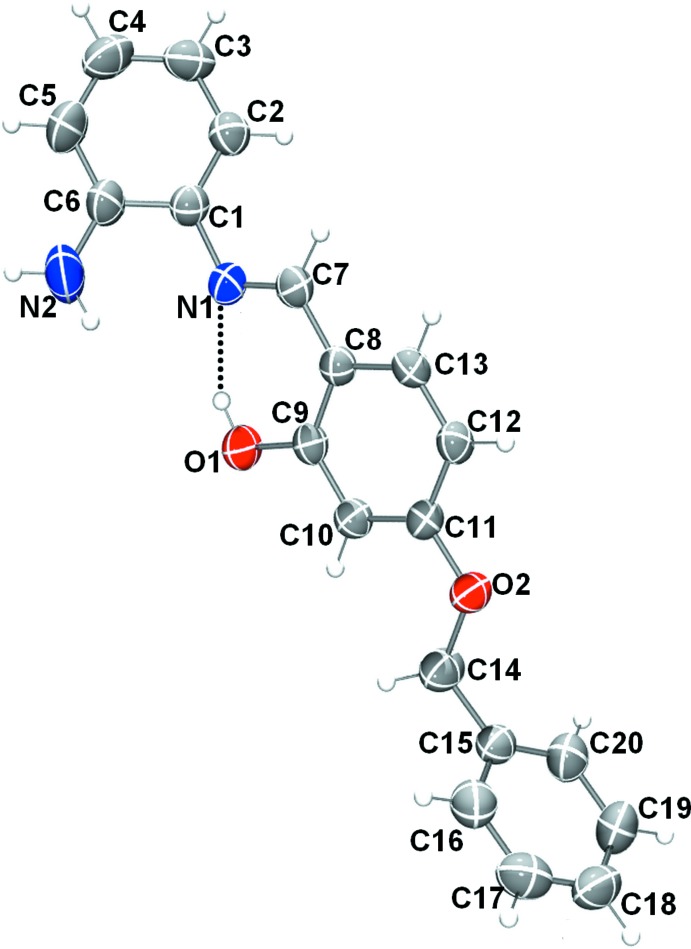
The mol­ecular structure of compound (I), with the atom labelling. Displacement ellipsoids are drawn at the 50% probability level. The intra­molecular O—H⋯N hydrogen bond is shown as a dashed line.

The mol­ecular structures of compounds (I) and (II), illus­trated in Figs. 1 ▸ and 2 ▸, respectively, are influenced by intra­molecular hydrogen bonds: the O—H⋯N hydrogen bond in (I) and the N—H⋯O contact in (II) (Tables 1 ▸ and 2 ▸) both form *S*(6) ring motifs. In compound (II), the N atom is protonated and the C9—O1 bond length, 1.277 (2) Å confirms this to be double bond. In compound (I), however, the C9=O1 bond length of 1.3498 (19) Å indicates a single bond. Bond C7=C8 [1.395 (3) Å] is a double bond in compound (II), whereas the corresponding bond in (I) [1.435 (3) Å] is a single bond. Compound (I) adopts the enol–imine tautomeric form and the configuration of the C7=N1 imine bond is *E* with a length of 1.288 (3) Å. In contrast the *o*-hy­droxy Schiff base of (II), has a *Z* configuration about the C7=C8 double bond and the mol­ecule adopts the keto–imine tautomeric form, with the N1—C7 bond length being 1.309 (2) Å. Neither mol­ecule is planar: in (I), the central ring (C8–C13) is inclined to the two outer rings (C1–C6 and C15–C20) by 46.80 (10) and 78.19 (10)°, respectively, while for (II), the dihedral angles between these rings are 5.11 (9) and 58.42 (11)°, respectively. In compound (II), the C1—N1—C7 angle is 127.15 (17)°.

## Supra­molecular features   

**Figure 3:**
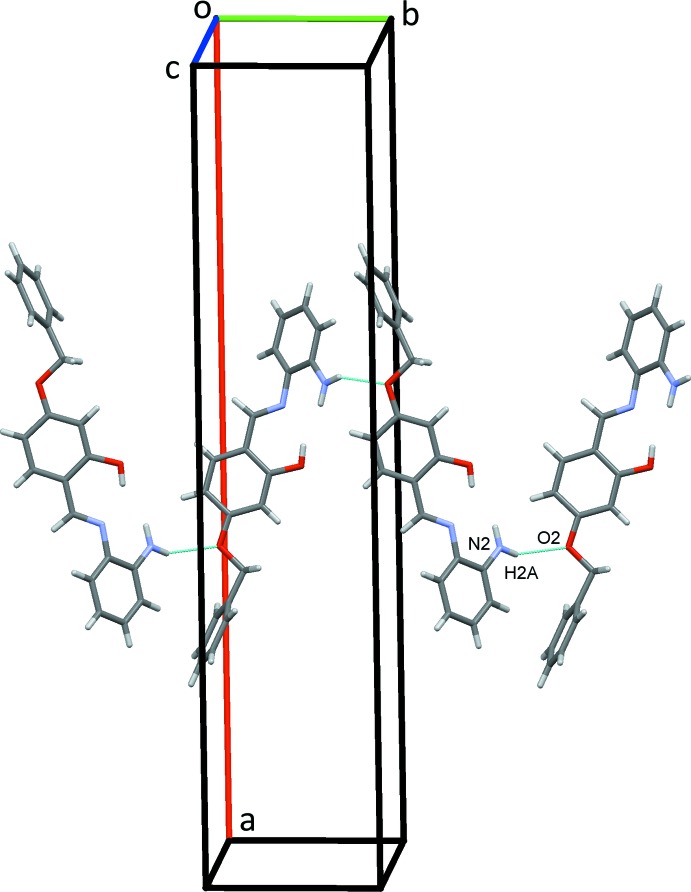
Zigzag chains of mol­ecules of (I) along the *b-*axis direction. Hydrogen bonds are drawn as blue dashed lines.

**Figure 4:**
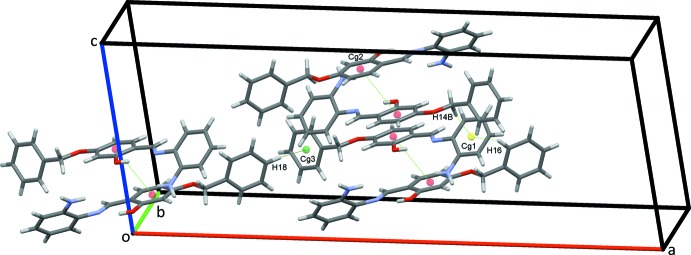
C—H⋯π and π–π conatcts (dotted green lines) in the crystal structure of (I).

**Figure 5:**
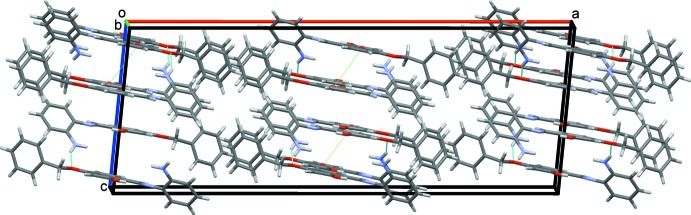
Overall packing for (I) viewed along the *b-*axis direction.

In the crystal of (I), strong N2—H2*A*⋯O2^i^ hydrogen bonds, Table 1 ▸, form zigzag chains of mol­ecules along the *b-*axis direction, Fig. 3 ▸. Weaker C—H⋯π and offset π–π stacking inter­actions also contribute to the packing (Fig. 4 ▸) [*Cg*2⋯*Cg*2(−*x*, *y*, −*z* + 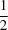) = 3.8151 (11) Å; *Cg*2 is the centroid of the central ring]. The overall crystal packing for this structure is shown in Fig. 5 ▸.

**Figure 6:**
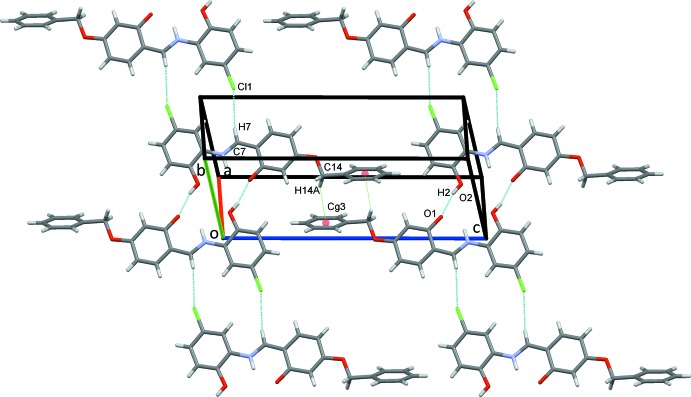
Sheets of mol­ecules of (II) parallel to (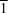
20).

**Figure 7:**
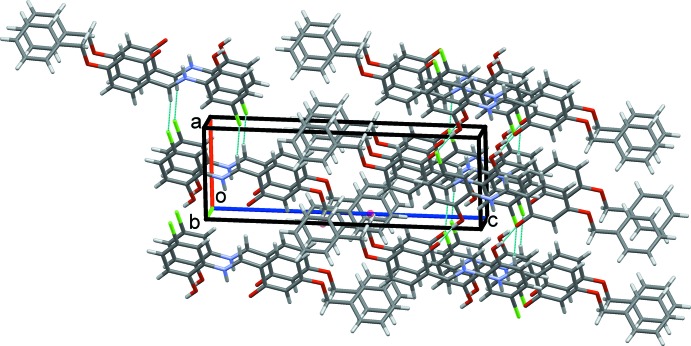
Overall packing for (II) viewed along the *b-*axis direction.

For (II), strong O2—H2⋯O1^i^ hydrogen bonds Table 2 ▸, form inversion dimers that enclose 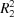
(18) rings. These combine with weaker C7—H7⋯Cl1 hydrogen bonds, which also generate inversion dimers but with 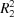
(14) motifs. Inversion-related C14—H14*A*⋯*Cg*3^ii^ contacts lead to the formation of sheets of mol­ecules parallel to (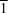
20), Fig. 6 ▸, which are stacked approximately along the *b-*axis direction. The overall packing for this structure is shown in Fig. 7 ▸.

## Database survey   

A search of the Cambridge Database (Version 5.39, updated February 2018; Groom *et al.* 2016 ▸) for structures similar to (I) gave two hits, *viz*. (*Z*)-6-{2-[(*E*)-2,4-di­hydroxy­benzyl­idene­amino]­phenyl­amino­methyl­ene}-3-hy­droxy­cyclo­hexa-2,4-dien­one (Fun *et al.*, 2008 ▸) and (*E*)-5-(benz­yloxy)-2-[(4-nitrophen­yl)carbonoimido­yl]phenol reported by us in 2015 (Ghichi *et al.*, 2015 ▸). More recently, we have described the very similar structure of (*E*)-5-benz­yloxy-2-{[(4-chloro­phen­yl)imino]meth­yl}phenol (Ghichi *et al.*, 2018 ▸). A search for analogues of (II) produced three related phenyl­ethyl­amino)­methyl­ene)cyclo­hexa-2,4-dien-1-ones (Chatziefthimiou *et al.*, 2006 ▸) and our recent contribution also reported (*E*)-5-benz­yloxy-2-({[2-(1*H*-indol-3-yl)eth­yl]iminium­yl)meth­yl)phen­ol­ate, which is closely similar to (II). The structures of Schiff bases derived from hydroxyaryl aldehydes have been the subject of a general survey, in which a number of structural errors, often involving misplaced H atoms, were pointed out (Blagus *et al.*, 2010 ▸).

## DFT-optimized calculations   

**Figure 8:**
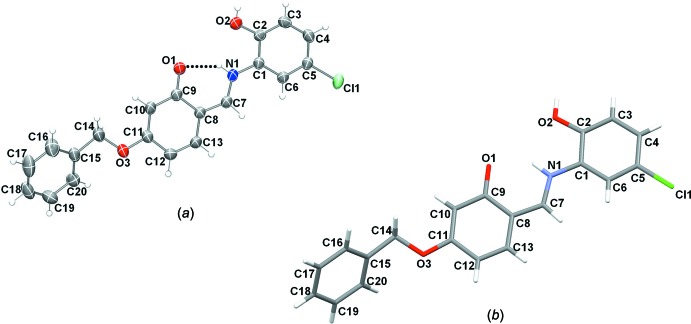
Comparison of the structures of (II) obtained from (*a*) the X-ray determination and (*b*) the DFT calculations.

**Figure 9:**
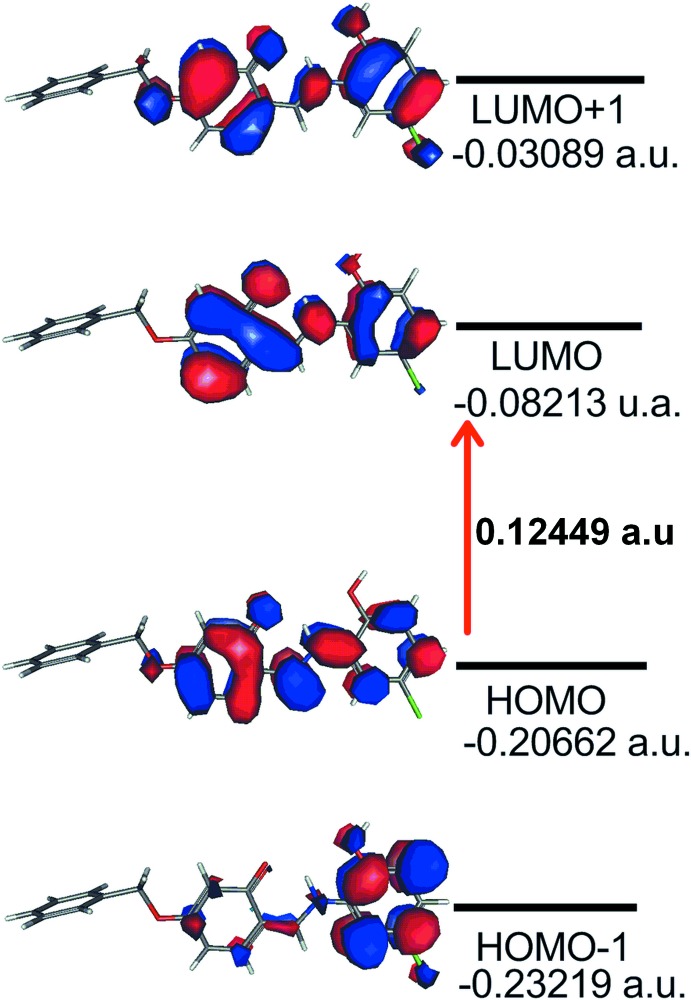
Electron distribution in the HOMO-1, HOMO, LUMO and LUMO-1 energy levels for (II).

DFT quantum chemical calculations were performed on mol­ecule (II) using the hybrid functional B3LYP (Becke *et al.*, 1993 ▸; Lee *et al.*, 1988 ▸), and base 6–311+G (*d*). The DFT structure optimization of (II) was performed starting from the X-ray geometry. The DFT and X-ray stuctures are compared in Fig. 8 ▸. The calculated values of bond lengths (Table 3 ▸) compare well with experimental values with the largest bond-length deviation being less than 0.031 Å from those found in the crystal structure. The adoption of the keto–imine tautomeric form is also predicted by these calculations. The study also shows that the HOMO and LUMO are localized in the plane extending from the chloro­hydroxy­benzene ring to the central phenol ring. The electron distribution of the HOMO-1, HOMO, LUMO and LUMO+1 energy levels is shown in Fig. 9 ▸. The occupied orbitals are predominantly of σ-character as is the LUMO, while LUMO+1 is mainly of π-character. The HOMO–LUMO gap is 0.12449 a.u, with frontier mol­ecular orbital energies, *E*_HOMO_ and *E*LUMO of −5.622 and −2.234 eV, respectively.

## Anti­oxidant activity   

The anti­oxidant activity profiles of (I) and (II) were determined using the copper(II)–neocuprine [Cu^II^–Nc] (CUPRAC) process (Apak *et al.*, 2004 ▸). The CUPRAC method (cupric ion reducing anti­oxidant capacity) follows the variation in the absorbance of the neocuproine (2,9-dimethyl-1,10-phenanthroline, Nc), copper^+2^ complex Nc_2_–Cu^+2^ In the presence of an anti­oxidant, the copper–neocuproine complex is reduced and this reaction is followed and qu­anti­fied spectrophotometrically at a wavelength of 450 nm. The results indicate that the percentage (%) inhibition (IC_50_) in the CUPRAC assay is small for both compounds in comparison to that for butyl­ated hy­droxy­toluene (BHT) that was used as a positive control. In Table 4 ▸ the values shown are the means of three separate measurements.

## Synthesis and crystallization   

**Compound (I)**

1,2-Di­amino­benzene (1 equiv.) and 4-benz­yloxy-2-hy­droxy­benzaldehyde (1 equiv.) in ethanol (15–20 ml) were refluxed for 1 h, the solvent was evaporated in *vacuo*. The residue was recrystallized from ethanol, yielding yellow block-like crystals on slow evaporation of the solvent. The purity of the compound was determined from its NMR spectrum (250 MHz, CDCl_3_). The azomethine proton appears in the 8.5–8.6 p.p.m.range, while the imine bond is characterized in the ^13^C NMR spectrum with the imine C and the C atom bound to the OH group appearing in the 161.58–163.20 p.p.m.range. ^1^H NMR: δ = 6.6–7.6 (*m*, 12H; *H-ar*), δ = 13.5 (*s*, 1H; *OH*), δ = 4 (*s*, 1H; *NH_2_*), δ = 5.1 (*s*, 1H; *CH_2_*–O). ^13^C NMR: 70.22, 127.66, 127.73, 128.32, 128.8, 140.66, 161.58, 163.02, 163.2.

**Compound (II)**

2-Amino-4-chloro­yphenol (1 equiv.) and 4-benz­yloxy-2-hy­droxy­benzaldehyde (1 equiv.) in ethanol (20 ml) were refluxed for 30–60 min, the solvent was evaporated in *vacuo*. The residue was recrystallized from ethanol, yielding orange block-like crystals on slow evaporation of the solvent. The purity of the compound was detemined by its NMR spectrum (250 MHz, CDCl_3_). ^1^H NMR: δ = 6.5–7.7 (*m*, 11H; *H-ar*), δ = 8.5–8.6 (*s*, 1H; *OH*), δ = 5.1 (*s*, 1H; *CH_2_*–O). ^13^C NMR: 55.6, 128.2, 128.7, 133.3, 136.4, 141.4, 159.69, 162.82, 163.77.

## Refinement   

Crystal data, data collection and structure refinement details are summarized in Table 5 ▸. In compound (I), the hydroxyl H atom was located in a difference-Fourier map and initially freely refined. In the final cycles of refinements it was positioned geometrically (O—H = 0.82 Å) and refined with *U*_iso_(H) = 1.5*U*_eq_(O). In compound (II), the H atoms on N1, C7 and O2 were located in a difference-Fourier and refined freely. For both compounds, the other C-bound H atoms were positioned geometrically (C—H = 0.97–0.97 Å) and refined as riding with *U*_iso_(H) = 1.2*U*_eq_(C).

## Supplementary Material

Crystal structure: contains datablock(s) global, I, II. DOI: 10.1107/S2056989018005662/sj5553sup1.cif

Structure factors: contains datablock(s) I. DOI: 10.1107/S2056989018005662/sj5553Isup2.hkl

Structure factors: contains datablock(s) II. DOI: 10.1107/S2056989018005662/sj5553IIsup3.hkl

Supporting information file. DOI: 10.1107/S2056989018005662/sj5553Isup4.cml

Supporting information file. DOI: 10.1107/S2056989018005662/sj5553IIsup5.cml

CCDC references: 1836250, 1836249

Additional supporting information:  crystallographic information; 3D view; checkCIF report

## Figures and Tables

**Figure 2:**
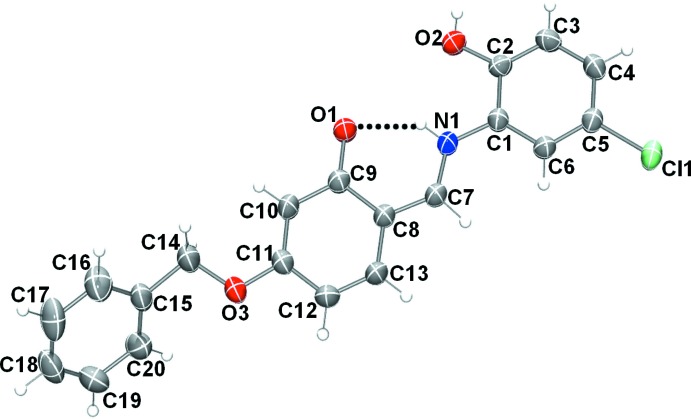
The mol­ecular structure of compound (II), with the atom labeling. Displacement ellipsoids are drawn at the 50% probability level. The intra­molecular N—H⋯O hydrogen bond is shown as a dashed line.

**Table 1:** Hydrogen-bond geometry (Å, °) for (I) *Cg*1 and *Cg*3 are the centroids of the C1–C6 and C15–C20 rings respectively.

*D*—H⋯*A*	*D*—H	H⋯*A*	*D*⋯*A*	*D*—H⋯*A*
O1—H1⋯N1	0.82	1.90	2.629 (2)	147
N2—H2*A*⋯O2^i^	0.86	2.43	3.211 (3)	151
C14—H14*B*⋯*Cg*1^ii^	0.97	2.74	3.704 (3)	171
C16—H16⋯*Cg*1^iii^	0.93	2.96	3.792 (3)	150
C18—H18⋯*Cg*3^iv^	0.93	2.94	3.620 (2)	131

Symmetry codes: (i) 
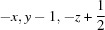
; (ii) 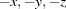; (iii) 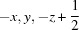; (iv) 
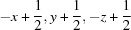
.

**Table 2:** Hydrogen-bond geometry (Å, °) for (II) *Cg*3 is the centroid of the C15–C20 ring.

*D*—H⋯*A*	*D*—H	H⋯*A*	*D*⋯*A*	*D*—H⋯*A*
N1—H1⋯O1	0.86 (2)	1.93 (2)	2.637 (2)	139 (2)
N1—H1⋯O2	0.86 (2)	2.27 (2)	2.620 (2)	104.5 (18)
O2—H2⋯O1^i^	0.80 (3)	1.84 (3)	2.619 (2)	165 (3)
C7—H7⋯Cl1^ii^	0.98 (2)	2.84 (2)	3.7971 (18)	164.5 (17)
C14—H14*A*⋯*Cg*3^iii^	0.97	2.71	3.569 (3)	148

Symmetry codes: (i) 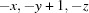; (ii) 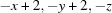; (iii) 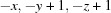.

**Table 3:** Experimental and calculated bond lengths (Å) for compound (II)

Bond	X-ray	B3LYP/6–311+G(*d*)
N1—C1	1.406 (2)	1.399
N1—C7	1.309 (2)	1.340
O1—C9	1.277 (2)	1.254
O2—C2	1.351 (2)	1.364
O3—C11	1.363 (2)	1.355
O3—C14	1.432 (3)	1.439
C1—C2	1.403 (2)	1.410
C1—C6	1.389 (2)	1.398
C2—C3	1.384 (3)	1.389
C3—C4	1.381 (3)	1.394
C5—C11	1.742 (2)	1.759
C7—C8	1.395 (3)	1.385
C9—C10	1.418 (3)	1.411
C10—C11	1.373 (3)	1.373
C12—C13	1.350 (3)	1.358
C14—C15	1.504 (3)	1.504
C16—C17	1.392 (4)	1.393
C19—C20	1.387 (3)	1.393

**Table 4:** Cupric ion reducing anti­oxidant capacity of compounds (I) and (II)

	Percentage (%) Inhibition							
	3.125 µg	6.25 µg	12.5 µg	25 µg	50 µg	100 µg	200 µg	A0.50 (μg/ml)
Compound (I)	0.28±0.01	0.46±0.00	0.76±0.03	1.55±0.04	2.60±0.14	3.81±0.15	4.33±0.04	7.4±0.21
Compound (II)	0.30±0.00	0.46±0.01	0.78±0.01	1.12±0.07	1.84±0.19	2.34±0.12	4.39±0.04	6.10±0.26
BHT	0.19±0.01	0.33±0.04	0.66±0.07	1.03±0.07	1.48±0.09	2.04±0.14	2.32±0.28	9.62±0.87

**Table 5:** Experimental details

	(I)	(II)
Crystal data		
Chemical formula	C_20_H_18_N_2_O_2_	C_20_H_16_ClNO_3_
*M*_r_	318.36	353.79
Crystal system, space group	Monoclinic, *C*2/*c*	Triclinic, *P* 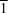
Temperature (K)	293	293
*a*, *b*, *c* (Å)	35.1343 (12), 7.2564 (2), 13.1450 (5)	5.9590 (2), 7.8710 (3), 17.9743 (6)
α, β, γ (°)	90, 95.553 (2), 90	98.381 (2), 93.817 (2), 90.294 (2)
*V* (Å^3^)	3335.57 (19)	832.11 (5)
*Z*	8	2
Radiation type	Mo *K*α	Mo *K*α
μ (mm^−1^)	0.08	0.25
Crystal size (mm)	0.03 × 0.02 × 0.01	0.03 × 0.02 × 0.01

Data collection		
Diffractometer	Bruker APEXII CCD	Bruker APEXII CCD
No. of measured, independent and observed [*I* > 2σ(*I*)] reflections	18218, 3811, 1915	13513, 3052, 2490
*R*_int_	0.072	0.025
(sin θ/λ)_max_ (Å^−1^)	0.650	0.606

Refinement		
*R*[*F* ^2^ > 2σ(*F* ^2^)], *wR*(*F* ^2^), *S*	0.049, 0.134, 1.00	0.042, 0.133, 1.10
No. of reflections	3811	3052
No. of parameters	221	238
H-atom treatment	H atoms treated by a mixture of independent and constrained refinement	H atoms treated by a mixture of independent and constrained refinement
Δρ_max_, Δρ_min_ (e Å^−3^)	0.17, −0.15	0.21, −0.21

Computer programs: *APEX2* and *SAINT* (Bruker, 2012 ▸), *SHELXS97* and *SHELXTL* (Sheldrick, 2008 ▸) and *SHELXL2017* (Sheldrick, 2015 ▸).

## supplementary crystallographic information

### (E)-2-{[(2-Aminophenyl)imino]methyl}-5-(benzyloxy)phenol (I) Crystal data

**Table:**

C_20_H_18_N_2_O_2_	*F*(000) = 1344
*M**_r_* = 318.36	*D*_x_ = 1.268 Mg m^−^^3^
Monoclinic, *C*2/*c*	Mo *K*α radiation, λ = 0.71073 Å
*a* = 35.1343 (12) Å	Cell parameters from 1907 reflections
*b* = 7.2564 (2) Å	θ = 2.9–21.9°
*c* = 13.1450 (5) Å	µ = 0.08 mm^−^^1^
β = 95.553 (2)°	*T* = 293 K
*V* = 3335.57 (19) Å^3^	Block, yellow
*Z* = 8	0.03 × 0.02 × 0.01 mm

### (E)-2-{[(2-Aminophenyl)imino]methyl}-5-(benzyloxy)phenol (I) Data collection

**Table:**

Bruker APEXII CCD diffractometer	*R*_int_ = 0.072
Detector resolution: 18.4 pixels mm^-1^	θ_max_ = 27.5°, θ_min_ = 3.4°
φ and ω scans	*h* = −45→45
18218 measured reflections	*k* = −9→9
3811 independent reflections	*l* = −16→17
1915 reflections with *I* > 2σ(*I*)	

### (E)-2-{[(2-Aminophenyl)imino]methyl}-5-(benzyloxy)phenol (I) Refinement

**Table:**

Refinement on *F*^2^	Primary atom site location: structure-invariant direct methods
Least-squares matrix: full	Secondary atom site location: difference Fourier map
*R*[*F*^2^ > 2σ(*F*^2^)] = 0.049	Hydrogen site location: mixed
*wR*(*F*^2^) = 0.134	H atoms treated by a mixture of independent and constrained refinement
*S* = 1.00	*w* = 1/[σ^2^(*F*_o_^2^) + (0.0527*P*)^2^ + 0.5669*P*] where *P* = (*F*_o_^2^ + 2*F*_c_^2^)/3
3811 reflections	(Δ/σ)_max_ = 0.001
221 parameters	Δρ_max_ = 0.17 e Å^−^^3^
0 restraints	Δρ_min_ = −0.15 e Å^−^^3^

### (E)-2-{[(2-Aminophenyl)imino]methyl}-5-(benzyloxy)phenol (I) Special details

**Table:**

Geometry. Bond distances, angles etc. have been calculated using the rounded fractional coordinates. All su's are estimated from the variances of the (full) variance-covariance matrix. The cell esds are taken into account in the estimation of distances, angles and torsion angles

### (E)-2-{[(2-Aminophenyl)imino]methyl}-5-(benzyloxy)phenol (I) Fractional atomic coordinates and isotropic or equivalent isotropic displacement parameters (Å^2^)

**Table:**

	*x*	*y*	*z*	*U*_iso_*/*U*_eq_	
O1	0.00836 (4)	−0.03108 (16)	0.12180 (11)	0.0578 (5)	
O2	0.10314 (4)	0.43746 (16)	0.17382 (10)	0.0513 (5)	
N1	−0.06331 (5)	0.0754 (2)	0.08936 (12)	0.0477 (6)	
N2	−0.09669 (6)	−0.1879 (3)	0.19901 (17)	0.0913 (9)	
C1	−0.10273 (6)	0.0392 (2)	0.06599 (15)	0.0458 (7)	
C2	−0.12530 (6)	0.1274 (3)	−0.01145 (17)	0.0529 (8)	
C3	−0.16347 (7)	0.0816 (3)	−0.03355 (19)	0.0653 (9)	
C4	−0.17934 (7)	−0.0518 (3)	0.0237 (2)	0.0708 (10)	
C5	−0.15720 (7)	−0.1400 (3)	0.1008 (2)	0.0683 (10)	
C6	−0.11880 (6)	−0.1003 (3)	0.12233 (17)	0.0553 (8)	
C7	−0.05090 (6)	0.2425 (3)	0.09311 (14)	0.0436 (7)	
C8	−0.01110 (5)	0.2870 (2)	0.11319 (14)	0.0396 (6)	
C9	0.01726 (6)	0.1499 (2)	0.12587 (14)	0.0411 (7)	
C10	0.05547 (6)	0.1960 (2)	0.14368 (15)	0.0452 (7)	
C11	0.06603 (5)	0.3795 (2)	0.15199 (14)	0.0410 (6)	
C12	0.03863 (6)	0.5185 (2)	0.13959 (14)	0.0431 (7)	
C13	0.00105 (6)	0.4708 (2)	0.12065 (14)	0.0421 (7)	
C14	0.13205 (6)	0.2969 (3)	0.18376 (19)	0.0617 (9)	
C15	0.16990 (6)	0.3854 (2)	0.21507 (18)	0.0490 (7)	
C16	0.18486 (7)	0.3842 (3)	0.3151 (2)	0.0623 (9)	
C17	0.22001 (7)	0.4634 (3)	0.3441 (2)	0.0693 (10)	
C18	0.24053 (7)	0.5423 (3)	0.2725 (2)	0.0698 (10)	
C19	0.22599 (7)	0.5434 (3)	0.1723 (2)	0.0715 (10)	
C20	0.19090 (6)	0.4654 (3)	0.14359 (19)	0.0617 (9)	
H1	−0.01490	−0.04310	0.11149	0.0870*	
H2	−0.11464	0.21904	−0.04924	0.0640*	
H2A	−0.10663	−0.27019	0.23540	0.1100*	
H2B	−0.07285	−0.16065	0.21082	0.1100*	
H3	−0.17822	0.14023	−0.08652	0.0780*	
H4	−0.20504	−0.08226	0.01022	0.0850*	
H5	−0.16836	−0.22861	0.13952	0.0820*	
H7	−0.0691 (5)	0.349 (3)	0.0810 (13)	0.045 (5)*	
H10	0.07401	0.10419	0.15006	0.0540*	
H12	0.04583	0.64178	0.14416	0.0520*	
H13	−0.01720	0.56385	0.11238	0.0510*	
H14A	0.12613	0.20744	0.23476	0.0740*	
H14B	0.13296	0.23331	0.11913	0.0740*	
H16	0.17121	0.32937	0.36425	0.0750*	
H17	0.22971	0.46292	0.41244	0.0830*	
H18	0.26425	0.59506	0.29182	0.0840*	
H19	0.23986	0.59698	0.12325	0.0860*	
H20	0.18125	0.46665	0.07518	0.0740*	

### (E)-2-{[(2-Aminophenyl)imino]methyl}-5-(benzyloxy)phenol (I) Atomic displacement parameters (Å^2^)

**Table:**

	*U*^11^	*U*^22^	*U*^33^	*U*^12^	*U*^13^	*U*^23^
O1	0.0526 (9)	0.0348 (7)	0.0852 (11)	−0.0032 (6)	0.0022 (8)	0.0001 (6)
O2	0.0397 (9)	0.0389 (7)	0.0745 (10)	0.0008 (6)	0.0021 (7)	−0.0048 (6)
N1	0.0457 (11)	0.0460 (10)	0.0507 (11)	−0.0054 (7)	0.0018 (8)	0.0016 (7)
N2	0.0912 (17)	0.0807 (15)	0.0962 (17)	−0.0325 (12)	−0.0211 (13)	0.0422 (12)
C1	0.0440 (13)	0.0420 (11)	0.0509 (13)	−0.0023 (9)	0.0028 (10)	−0.0036 (9)
C2	0.0527 (15)	0.0473 (11)	0.0581 (14)	0.0000 (9)	0.0020 (11)	0.0007 (10)
C3	0.0532 (16)	0.0590 (14)	0.0801 (18)	0.0082 (11)	−0.0113 (13)	−0.0071 (12)
C4	0.0454 (15)	0.0639 (15)	0.102 (2)	−0.0072 (11)	0.0012 (14)	−0.0144 (14)
C5	0.0598 (17)	0.0602 (14)	0.0848 (19)	−0.0201 (11)	0.0058 (14)	0.0032 (12)
C6	0.0544 (15)	0.0470 (12)	0.0633 (15)	−0.0100 (10)	−0.0002 (12)	0.0042 (10)
C7	0.0474 (13)	0.0430 (11)	0.0403 (12)	0.0003 (9)	0.0041 (9)	0.0017 (8)
C8	0.0417 (12)	0.0403 (10)	0.0365 (11)	−0.0009 (8)	0.0027 (9)	0.0003 (8)
C9	0.0476 (13)	0.0347 (10)	0.0411 (12)	−0.0006 (8)	0.0056 (10)	−0.0004 (8)
C10	0.0434 (13)	0.0369 (10)	0.0553 (13)	0.0036 (8)	0.0043 (10)	−0.0015 (9)
C11	0.0401 (12)	0.0418 (10)	0.0415 (11)	−0.0024 (8)	0.0056 (9)	−0.0018 (8)
C12	0.0471 (13)	0.0344 (10)	0.0472 (12)	−0.0014 (8)	0.0021 (10)	−0.0014 (8)
C13	0.0457 (13)	0.0380 (10)	0.0422 (12)	0.0044 (8)	0.0021 (9)	0.0006 (8)
C14	0.0454 (14)	0.0441 (12)	0.0954 (19)	0.0041 (9)	0.0055 (12)	−0.0047 (11)
C15	0.0398 (13)	0.0381 (10)	0.0683 (15)	0.0037 (8)	0.0017 (11)	0.0001 (9)
C16	0.0557 (16)	0.0601 (14)	0.0720 (18)	0.0053 (11)	0.0111 (13)	0.0067 (11)
C17	0.0664 (18)	0.0707 (15)	0.0673 (17)	0.0101 (13)	−0.0113 (14)	−0.0064 (12)
C18	0.0473 (15)	0.0571 (14)	0.102 (2)	−0.0037 (11)	−0.0074 (15)	−0.0060 (13)
C19	0.0623 (17)	0.0643 (15)	0.088 (2)	−0.0158 (12)	0.0079 (15)	0.0094 (13)
C20	0.0618 (16)	0.0554 (13)	0.0668 (16)	−0.0088 (11)	0.0014 (13)	0.0057 (11)

### (E)-2-{[(2-Aminophenyl)imino]methyl}-5-(benzyloxy)phenol (I) Geometric parameters (Å, º)

**Table:**

O1—C9	1.3498 (19)	C14—C15	1.498 (3)
O2—C11	1.374 (2)	C15—C20	1.378 (3)
O2—C14	1.437 (3)	C15—C16	1.368 (3)
N1—C1	1.414 (3)	C16—C17	1.382 (3)
N1—C7	1.288 (3)	C17—C18	1.366 (4)
O1—H1	0.8200	C18—C19	1.366 (4)
N2—C6	1.368 (3)	C19—C20	1.376 (3)
C1—C2	1.385 (3)	C2—H2	0.9300
C1—C6	1.405 (3)	C3—H3	0.9300
N2—H2A	0.8600	C4—H4	0.9300
N2—H2B	0.8600	C5—H5	0.9300
C2—C3	1.385 (3)	C7—H7	1.01 (2)
C3—C4	1.377 (3)	C10—H10	0.9300
C4—C5	1.375 (4)	C12—H12	0.9300
C5—C6	1.382 (3)	C13—H13	0.9300
C7—C8	1.435 (3)	C14—H14A	0.9700
C8—C9	1.407 (2)	C14—H14B	0.9700
C8—C13	1.401 (2)	C16—H16	0.9300
C9—C10	1.381 (3)	C17—H17	0.9300
C10—C11	1.384 (2)	C18—H18	0.9300
C11—C12	1.393 (2)	C19—H19	0.9300
C12—C13	1.364 (3)	C20—H20	0.9300
			
C11—O2—C14	116.75 (13)	C17—C18—C19	119.6 (2)
C1—N1—C7	120.27 (16)	C18—C19—C20	120.3 (2)
C9—O1—H1	109.00	C15—C20—C19	120.7 (2)
N1—C1—C2	123.57 (17)	C1—C2—H2	119.00
C2—C1—C6	119.33 (19)	C3—C2—H2	119.00
N1—C1—C6	117.04 (17)	C2—C3—H3	120.00
H2A—N2—H2B	120.00	C4—C3—H3	120.00
C6—N2—H2B	120.00	C3—C4—H4	120.00
C1—C2—C3	121.1 (2)	C5—C4—H4	120.00
C6—N2—H2A	120.00	C4—C5—H5	119.00
C2—C3—C4	119.3 (2)	C6—C5—H5	119.00
C3—C4—C5	120.0 (2)	N1—C7—H7	120.6 (11)
C4—C5—C6	121.7 (2)	C8—C7—H7	116.7 (11)
N2—C6—C5	121.8 (2)	C9—C10—H10	120.00
C1—C6—C5	118.5 (2)	C11—C10—H10	120.00
N2—C6—C1	119.7 (2)	C11—C12—H12	121.00
N1—C7—C8	122.64 (19)	C13—C12—H12	121.00
C7—C8—C9	121.96 (15)	C8—C13—H13	119.00
C7—C8—C13	120.81 (17)	C12—C13—H13	119.00
C9—C8—C13	117.23 (17)	O2—C14—H14A	110.00
O1—C9—C10	117.38 (16)	O2—C14—H14B	110.00
C8—C9—C10	120.97 (14)	C15—C14—H14A	110.00
O1—C9—C8	121.65 (17)	C15—C14—H14B	110.00
C9—C10—C11	119.68 (16)	H14A—C14—H14B	108.00
C10—C11—C12	120.70 (17)	C15—C16—H16	120.00
O2—C11—C10	123.57 (15)	C17—C16—H16	120.00
O2—C11—C12	115.72 (14)	C16—C17—H17	120.00
C11—C12—C13	118.90 (14)	C18—C17—H17	120.00
C8—C13—C12	122.50 (16)	C17—C18—H18	120.00
O2—C14—C15	108.77 (16)	C19—C18—H18	120.00
C14—C15—C16	120.6 (2)	C18—C19—H19	120.00
C14—C15—C20	120.9 (2)	C20—C19—H19	120.00
C16—C15—C20	118.5 (2)	C15—C20—H20	120.00
C15—C16—C17	120.8 (2)	C19—C20—H20	120.00
C16—C17—C18	120.1 (2)		
			
C14—O2—C11—C10	2.8 (3)	C13—C8—C9—O1	178.84 (17)
C14—O2—C11—C12	−178.09 (17)	C13—C8—C9—C10	−0.8 (3)
C11—O2—C14—C15	−175.82 (17)	C7—C8—C13—C12	−179.96 (18)
C7—N1—C1—C2	44.0 (3)	C9—C8—C13—C12	−0.1 (3)
C7—N1—C1—C6	−139.06 (19)	O1—C9—C10—C11	−177.83 (17)
C1—N1—C7—C8	−177.94 (17)	C8—C9—C10—C11	1.8 (3)
N1—C1—C2—C3	177.54 (19)	C9—C10—C11—O2	177.20 (17)
C6—C1—C2—C3	0.6 (3)	C9—C10—C11—C12	−1.9 (3)
N1—C1—C6—N2	3.0 (3)	O2—C11—C12—C13	−178.16 (16)
N1—C1—C6—C5	−179.52 (19)	C10—C11—C12—C13	1.0 (3)
C2—C1—C6—N2	−179.9 (2)	C11—C12—C13—C8	0.0 (3)
C2—C1—C6—C5	−2.4 (3)	O2—C14—C15—C16	99.2 (2)
C1—C2—C3—C4	1.1 (3)	O2—C14—C15—C20	−82.5 (2)
C2—C3—C4—C5	−1.0 (4)	C14—C15—C16—C17	179.1 (2)
C3—C4—C5—C6	−0.8 (4)	C20—C15—C16—C17	0.8 (3)
C4—C5—C6—N2	−180.0 (2)	C14—C15—C20—C19	−178.73 (19)
C4—C5—C6—C1	2.5 (3)	C16—C15—C20—C19	−0.4 (3)
N1—C7—C8—C9	2.8 (3)	C15—C16—C17—C18	−0.8 (3)
N1—C7—C8—C13	−177.43 (18)	C16—C17—C18—C19	0.4 (3)
C7—C8—C9—O1	−1.4 (3)	C17—C18—C19—C20	−0.1 (3)
C7—C8—C9—C10	179.05 (18)	C18—C19—C20—C15	0.1 (3)

### (E)-2-{[(2-Aminophenyl)imino]methyl}-5-(benzyloxy)phenol (I) Hydrogen-bond geometry (Å, º)

Cg1 and Cg3 are the centroids of the C1–C6 and C15–C20 rings respectively.

**Table:**

*D*—H···*A*	*D*—H	H···*A*	*D*···*A*	*D*—H···*A*
O1—H1···N1	0.82	1.90	2.629 (2)	147
N2—H2*A*···O2^i^	0.86	2.43	3.211 (3)	151
C14—H14*B*···*Cg*1^ii^	0.97	2.74	3.704 (3)	171
C16—H16···*Cg*1^iii^	0.93	2.96	3.792 (3)	150
C18—H18···*Cg*3^iv^	0.93	2.94	3.620 (2)	131

Symmetry codes: (i) −*x*, *y*−1, −*z*+1/2; (ii) −*x*, −*y*, −*z*; (iii) −*x*, *y*, −*z*+1/2; (iv) −*x*+1/2, *y*+1/2, −*z*+1/2.

### (Z)-3-Benzyloxy-6-{[(5-chloro-2-hydroxyphenyl)amino]methylidene}cyclohexa-2,4-dien-1-one (II) Crystal data

**Table:**

C_20_H_16_ClNO_3_	*Z* = 2
*M**_r_* = 353.79	*F*(000) = 368
Triclinic, *P*1	*D*_x_ = 1.412 Mg m^−^^3^
*a* = 5.9590 (2) Å	Mo *K*α radiation, λ = 0.71073 Å
*b* = 7.8710 (3) Å	Cell parameters from 5281 reflections
*c* = 17.9743 (6) Å	θ = 2.7–30.7°
α = 98.381 (2)°	µ = 0.25 mm^−^^1^
β = 93.817 (2)°	*T* = 293 K
γ = 90.294 (2)°	Block, orange
*V* = 832.11 (5) Å^3^	0.03 × 0.02 × 0.01 mm

### (Z)-3-Benzyloxy-6-{[(5-chloro-2-hydroxyphenyl)amino]methylidene}cyclohexa-2,4-dien-1-one (II) Data collection

**Table:**

Bruker APEXII CCD diffractometer	*R*_int_ = 0.025
Detector resolution: 18.4 pixels mm^-1^	θ_max_ = 25.5°, θ_min_ = 2.6°
φ and ω scans	*h* = −6→7
13513 measured reflections	*k* = −9→9
3052 independent reflections	*l* = −21→21
2490 reflections with *I* > 2σ(*I*)	

### (Z)-3-Benzyloxy-6-{[(5-chloro-2-hydroxyphenyl)amino]methylidene}cyclohexa-2,4-dien-1-one (II) Refinement

**Table:**

Refinement on *F*^2^	Primary atom site location: structure-invariant direct methods
Least-squares matrix: full	Secondary atom site location: difference Fourier map
*R*[*F*^2^ > 2σ(*F*^2^)] = 0.042	Hydrogen site location: mixed
*wR*(*F*^2^) = 0.133	H atoms treated by a mixture of independent and constrained refinement
*S* = 1.10	*w* = 1/[σ^2^(*F*_o_^2^) + (0.0727*P*)^2^ + 0.2078*P*] where *P* = (*F*_o_^2^ + 2*F*_c_^2^)/3
3052 reflections	(Δ/σ)_max_ < 0.001
238 parameters	Δρ_max_ = 0.21 e Å^−^^3^
0 restraints	Δρ_min_ = −0.21 e Å^−^^3^

### (Z)-3-Benzyloxy-6-{[(5-chloro-2-hydroxyphenyl)amino]methylidene}cyclohexa-2,4-dien-1-one (II) Special details

**Table:**

Geometry. Bond distances, angles etc. have been calculated using the rounded fractional coordinates. All su's are estimated from the variances of the (full) variance-covariance matrix. The cell esds are taken into account in the estimation of distances, angles and torsion angles

### (Z)-3-Benzyloxy-6-{[(5-chloro-2-hydroxyphenyl)amino]methylidene}cyclohexa-2,4-dien-1-one (II) Fractional atomic coordinates and isotropic or equivalent isotropic displacement parameters (Å^2^)

**Table:**

	*x*	*y*	*z*	*U*_iso_*/*U*_eq_	
Cl1	0.93492 (8)	0.93423 (7)	−0.13197 (3)	0.0533 (2)	
O1	0.1309 (2)	0.6289 (2)	0.14736 (7)	0.0518 (5)	
O2	0.1581 (3)	0.5583 (2)	−0.05318 (9)	0.0546 (5)	
O3	0.3158 (2)	0.7742 (2)	0.41212 (7)	0.0520 (5)	
N1	0.4214 (3)	0.7241 (2)	0.05641 (8)	0.0382 (5)	
C1	0.4780 (3)	0.7305 (2)	−0.01782 (10)	0.0348 (5)	
C2	0.3343 (3)	0.6417 (2)	−0.07572 (10)	0.0381 (6)	
C3	0.3805 (3)	0.6429 (3)	−0.15011 (10)	0.0444 (6)	
C4	0.5643 (3)	0.7329 (3)	−0.16776 (10)	0.0433 (6)	
C5	0.7028 (3)	0.8202 (2)	−0.11011 (10)	0.0374 (6)	
C6	0.6635 (3)	0.8205 (2)	−0.03551 (10)	0.0371 (5)	
C7	0.5388 (3)	0.7860 (2)	0.11873 (10)	0.0394 (6)	
C8	0.4718 (3)	0.7766 (2)	0.19092 (10)	0.0380 (5)	
C9	0.2627 (3)	0.6945 (2)	0.20317 (10)	0.0380 (5)	
C10	0.2119 (3)	0.6906 (3)	0.27886 (10)	0.0426 (6)	
C11	0.3535 (3)	0.7669 (3)	0.33778 (10)	0.0409 (6)	
C12	0.5562 (3)	0.8502 (3)	0.32557 (11)	0.0481 (7)	
C13	0.6123 (3)	0.8526 (3)	0.25418 (11)	0.0465 (6)	
C14	0.1117 (4)	0.6974 (3)	0.43003 (11)	0.0572 (8)	
C15	0.1044 (4)	0.7217 (3)	0.51439 (10)	0.0474 (7)	
C16	−0.0740 (4)	0.8010 (3)	0.54911 (14)	0.0632 (8)	
C17	−0.0843 (5)	0.8143 (3)	0.62686 (15)	0.0719 (9)	
C18	0.0817 (5)	0.7470 (3)	0.66975 (12)	0.0631 (8)	
C19	0.2599 (5)	0.6690 (4)	0.63599 (13)	0.0677 (9)	
C20	0.2723 (4)	0.6576 (3)	0.55862 (12)	0.0621 (8)	
H1	0.299 (4)	0.674 (3)	0.0639 (13)	0.057 (7)*	
H2	0.080 (5)	0.509 (4)	−0.0877 (18)	0.086 (10)*	
H3	0.28678	0.58262	−0.18855	0.0530*	
H4	0.59415	0.73465	−0.21778	0.0520*	
H6	0.75959	0.88004	0.00245	0.0450*	
H7	0.686 (4)	0.840 (3)	0.1147 (11)	0.043 (5)*	
H10	0.08097	0.63559	0.28872	0.0510*	
H12	0.64933	0.90248	0.36612	0.0580*	
H13	0.74667	0.90542	0.24615	0.0560*	
H14A	0.10695	0.57595	0.41018	0.0690*	
H14B	−0.01698	0.75127	0.40768	0.0690*	
H16	−0.18852	0.84600	0.52046	0.0760*	
H17	−0.20480	0.86928	0.64988	0.0860*	
H18	0.07282	0.75461	0.72159	0.0760*	
H19	0.37329	0.62325	0.66483	0.0810*	
H20	0.39585	0.60591	0.53619	0.0740*	

### (Z)-3-Benzyloxy-6-{[(5-chloro-2-hydroxyphenyl)amino]methylidene}cyclohexa-2,4-dien-1-one (II) Atomic displacement parameters (Å^2^)

**Table:**

	*U*^11^	*U*^22^	*U*^33^	*U*^12^	*U*^13^	*U*^23^
Cl1	0.0448 (3)	0.0693 (4)	0.0476 (3)	−0.0148 (2)	0.0136 (2)	0.0105 (2)
O1	0.0473 (8)	0.0697 (10)	0.0352 (7)	−0.0287 (7)	−0.0005 (6)	−0.0002 (6)
O2	0.0469 (8)	0.0723 (11)	0.0437 (8)	−0.0303 (8)	−0.0049 (7)	0.0105 (7)
O3	0.0513 (8)	0.0735 (10)	0.0313 (7)	−0.0160 (7)	0.0047 (6)	0.0075 (6)
N1	0.0356 (8)	0.0456 (9)	0.0330 (8)	−0.0118 (7)	0.0042 (6)	0.0039 (6)
C1	0.0346 (9)	0.0376 (9)	0.0323 (9)	−0.0039 (7)	0.0041 (7)	0.0046 (7)
C2	0.0340 (9)	0.0399 (10)	0.0397 (10)	−0.0075 (8)	−0.0029 (8)	0.0066 (7)
C3	0.0469 (11)	0.0494 (11)	0.0349 (10)	−0.0082 (9)	−0.0067 (8)	0.0037 (8)
C4	0.0487 (11)	0.0505 (11)	0.0310 (9)	−0.0036 (9)	0.0038 (8)	0.0068 (8)
C5	0.0340 (9)	0.0404 (10)	0.0385 (10)	−0.0029 (8)	0.0062 (7)	0.0066 (7)
C6	0.0343 (9)	0.0421 (10)	0.0335 (9)	−0.0077 (8)	0.0011 (7)	0.0017 (7)
C7	0.0352 (10)	0.0454 (11)	0.0373 (10)	−0.0108 (8)	0.0025 (8)	0.0052 (8)
C8	0.0353 (9)	0.0440 (10)	0.0345 (9)	−0.0087 (8)	0.0025 (7)	0.0051 (7)
C9	0.0370 (9)	0.0407 (10)	0.0352 (9)	−0.0089 (8)	0.0021 (7)	0.0028 (7)
C10	0.0388 (10)	0.0513 (11)	0.0379 (10)	−0.0134 (8)	0.0057 (8)	0.0063 (8)
C11	0.0426 (10)	0.0484 (11)	0.0321 (9)	−0.0043 (8)	0.0034 (8)	0.0068 (8)
C12	0.0414 (11)	0.0641 (13)	0.0370 (10)	−0.0152 (9)	−0.0041 (8)	0.0047 (9)
C13	0.0376 (10)	0.0631 (13)	0.0380 (10)	−0.0178 (9)	0.0004 (8)	0.0066 (9)
C14	0.0551 (13)	0.0804 (16)	0.0358 (10)	−0.0195 (11)	0.0051 (9)	0.0072 (10)
C15	0.0520 (12)	0.0559 (12)	0.0340 (10)	−0.0134 (9)	0.0084 (9)	0.0035 (8)
C16	0.0670 (15)	0.0686 (15)	0.0584 (14)	0.0097 (12)	0.0141 (12)	0.0195 (11)
C17	0.0911 (19)	0.0635 (15)	0.0647 (15)	0.0071 (14)	0.0405 (15)	0.0050 (12)
C18	0.0877 (18)	0.0656 (15)	0.0342 (10)	−0.0144 (13)	0.0125 (11)	−0.0020 (10)
C19	0.0683 (16)	0.0918 (19)	0.0397 (12)	−0.0086 (14)	−0.0080 (11)	0.0046 (11)
C20	0.0496 (13)	0.0896 (18)	0.0434 (12)	0.0007 (12)	0.0051 (10)	−0.0033 (11)

### (Z)-3-Benzyloxy-6-{[(5-chloro-2-hydroxyphenyl)amino]methylidene}cyclohexa-2,4-dien-1-one (II) Geometric parameters (Å, º)

**Table:**

Cl1—C5	1.7423 (18)	C14—C15	1.504 (3)
O1—C9	1.277 (2)	C15—C20	1.380 (3)
O2—C2	1.351 (2)	C15—C16	1.375 (3)
O3—C11	1.363 (2)	C16—C17	1.392 (4)
O3—C14	1.432 (3)	C17—C18	1.370 (4)
N1—C1	1.406 (2)	C18—C19	1.363 (4)
N1—C7	1.309 (2)	C19—C20	1.387 (3)
O2—H2	0.80 (3)	C3—H3	0.9300
C1—C2	1.403 (2)	C4—H4	0.9300
C1—C6	1.389 (2)	C6—H6	0.9300
N1—H1	0.86 (2)	C7—H7	0.98 (2)
C2—C3	1.384 (3)	C10—H10	0.9300
C3—C4	1.381 (3)	C12—H12	0.9300
C4—C5	1.378 (3)	C13—H13	0.9300
C5—C6	1.376 (3)	C14—H14A	0.9700
C7—C8	1.395 (3)	C14—H14B	0.9700
C8—C13	1.422 (3)	C16—H16	0.9300
C8—C9	1.445 (2)	C17—H17	0.9300
C9—C10	1.418 (3)	C18—H18	0.9300
C10—C11	1.373 (3)	C19—H19	0.9300
C11—C12	1.416 (3)	C20—H20	0.9300
C12—C13	1.350 (3)		
			
C11—O3—C14	117.35 (14)	C15—C16—C17	120.4 (2)
C1—N1—C7	127.15 (17)	C16—C17—C18	120.5 (2)
C2—O2—H2	113 (2)	C17—C18—C19	119.6 (2)
N1—C1—C6	123.47 (16)	C18—C19—C20	120.1 (2)
C2—C1—C6	119.86 (16)	C15—C20—C19	121.1 (2)
N1—C1—C2	116.67 (16)	C2—C3—H3	120.00
C1—N1—H1	119.4 (15)	C4—C3—H3	120.00
C7—N1—H1	113.4 (15)	C3—C4—H4	121.00
C1—C2—C3	119.57 (16)	C5—C4—H4	121.00
O2—C2—C3	124.73 (17)	C1—C6—H6	121.00
O2—C2—C1	115.70 (16)	C5—C6—H6	121.00
C2—C3—C4	120.61 (17)	N1—C7—H7	118.2 (12)
C3—C4—C5	118.97 (17)	C8—C7—H7	117.5 (12)
Cl1—C5—C4	119.22 (14)	C9—C10—H10	120.00
Cl1—C5—C6	118.73 (13)	C11—C10—H10	120.00
C4—C5—C6	122.05 (17)	C11—C12—H12	121.00
C1—C6—C5	118.93 (16)	C13—C12—H12	120.00
N1—C7—C8	124.28 (17)	C8—C13—H13	119.00
C9—C8—C13	119.22 (16)	C12—C13—H13	119.00
C7—C8—C9	121.97 (16)	O3—C14—H14A	110.00
C7—C8—C13	118.80 (16)	O3—C14—H14B	110.00
O1—C9—C8	120.48 (16)	C15—C14—H14A	110.00
O1—C9—C10	122.15 (16)	C15—C14—H14B	110.00
C8—C9—C10	117.36 (16)	H14A—C14—H14B	108.00
C9—C10—C11	120.87 (17)	C15—C16—H16	120.00
C10—C11—C12	121.61 (17)	C17—C16—H16	120.00
O3—C11—C12	113.29 (16)	C16—C17—H17	120.00
O3—C11—C10	125.09 (17)	C18—C17—H17	120.00
C11—C12—C13	119.03 (18)	C17—C18—H18	120.00
C8—C13—C12	121.89 (18)	C19—C18—H18	120.00
O3—C14—C15	108.26 (17)	C18—C19—H19	120.00
C14—C15—C16	121.1 (2)	C20—C19—H19	120.00
C14—C15—C20	120.6 (2)	C15—C20—H20	119.00
C16—C15—C20	118.32 (19)	C19—C20—H20	119.00
			
C14—O3—C11—C10	−0.5 (3)	C7—C8—C9—C10	179.38 (17)
C14—O3—C11—C12	178.36 (19)	C13—C8—C9—O1	178.32 (17)
C11—O3—C14—C15	−179.52 (18)	C13—C8—C9—C10	−1.6 (3)
C7—N1—C1—C2	174.54 (17)	C7—C8—C13—C12	179.1 (2)
C7—N1—C1—C6	−6.3 (3)	C9—C8—C13—C12	0.1 (3)
C1—N1—C7—C8	179.41 (17)	O1—C9—C10—C11	−177.95 (19)
N1—C1—C2—O2	−0.6 (2)	C8—C9—C10—C11	2.0 (3)
N1—C1—C2—C3	180.00 (18)	C9—C10—C11—O3	177.97 (19)
C6—C1—C2—O2	−179.83 (16)	C9—C10—C11—C12	−0.8 (3)
C6—C1—C2—C3	0.8 (3)	O3—C11—C12—C13	−179.7 (2)
N1—C1—C6—C5	−179.28 (16)	C10—C11—C12—C13	−0.8 (3)
C2—C1—C6—C5	−0.2 (2)	C11—C12—C13—C8	1.2 (3)
O2—C2—C3—C4	179.61 (19)	O3—C14—C15—C16	124.1 (2)
C1—C2—C3—C4	−1.1 (3)	O3—C14—C15—C20	−58.9 (3)
C2—C3—C4—C5	0.7 (3)	C14—C15—C16—C17	176.5 (2)
C3—C4—C5—Cl1	−179.94 (17)	C20—C15—C16—C17	−0.5 (4)
C3—C4—C5—C6	0.0 (3)	C14—C15—C20—C19	−175.6 (2)
Cl1—C5—C6—C1	179.67 (13)	C16—C15—C20—C19	1.4 (4)
C4—C5—C6—C1	−0.2 (3)	C15—C16—C17—C18	−0.7 (4)
N1—C7—C8—C9	0.5 (3)	C16—C17—C18—C19	1.1 (4)
N1—C7—C8—C13	−178.51 (18)	C17—C18—C19—C20	−0.2 (4)
C7—C8—C9—O1	−0.7 (3)	C18—C19—C20—C15	−1.1 (4)

### (Z)-3-Benzyloxy-6-{[(5-chloro-2-hydroxyphenyl)amino]methylidene}cyclohexa-2,4-dien-1-one (II) Hydrogen-bond geometry (Å, º)

Cg3 is the centroid of the C15–C20 ring.

**Table:**

*D*—H···*A*	*D*—H	H···*A*	*D*···*A*	*D*—H···*A*
N1—H1···O1	0.86 (2)	1.93 (2)	2.637 (2)	139 (2)
N1—H1···O2	0.86 (2)	2.27 (2)	2.620 (2)	104.5 (18)
O2—H2···O1^i^	0.80 (3)	1.84 (3)	2.619 (2)	165 (3)
C7—H7···Cl1^ii^	0.98 (2)	2.84 (2)	3.7971 (18)	164.5 (17)
C14—H14*A*···*Cg*3^iii^	0.97	2.71	3.569 (3)	148

Symmetry codes: (i) −*x*, −*y*+1, −*z*; (ii) −*x*+2, −*y*+2, −*z*; (iii) −*x*, −*y*+1, −*z*+1.
